# Personalized Medicine for Rheumatic Diseases: Bridging Laboratory, Imaging, and Clinimetric Tools for Tailored Diagnosis and Treatment

**DOI:** 10.3390/jpm16100496

**Published:** 2026-09-24

**Authors:** Rossella De Angelis

**Affiliations:** 1 Rheumatology Unit, Department of Clinical and Molecular Sciences, Polytechnic University of Marche, 60121 Ancona, Italy; r.deangelis@staff.univpm.it; 2 “Carlo Urbani” Hospital, Via Aldo Moro, 25, 60035 Jesi, Italy

The aim of launching this Special Issue was to bring together contributions on recent findings in rheumatic diseases, with a particular focus on the methodologies and tools used to detect, diagnose, and manage their clinical counterparts. We hoped to highlight the value of multidisciplinary collaboration—drawing on the expertise of laboratory, clinical, imaging, and clinimetric researchers—to help fill existing knowledge gaps in this field. Five papers were ultimately published in this collection, and while they span a broad range of conditions and clinical settings, they converge on a common thread: each demonstrates how a specific diagnostic or clinimetric tool can be used to personalize a clinical decision, whether diagnostic or therapeutic, in a rheumatic disease.

Personalized medicine has become a central paradigm in rheumatology over the past two to three years, driven by parallel advances in treatment recommendations, classification tools, and diagnostic technology. In rheumatoid arthritis (RA), the 2025 update of the EULAR management recommendations explicitly stratifies treatment escalation according to individual prognostic factors—including autoantibody status, acute-phase reactants, and disease activity—rather than a uniform treat-to-target algorithm [1], building on the earlier EULAR points to consider for difficult-to-treat RA, which formalized the recognition that a substantial minority of patients require a dedicated, individualized diagnostic and therapeutic pathway [2]. For the first time, the 2023 ACR/CHEST guideline for the screening and monitoring of interstitial lung disease in systemic sclerosis (SSc) has harmonized recommendations on which non-invasive tools should be used and at what interval, explicitly acknowledging the heterogeneity of individual risk [3]. In ANCA-associated vasculitis, the 2024 KDIGO guideline and a parallel update of treatment standards have reinforced the role of kidney biopsy not merely as a confirmatory diagnostic test but as a source of prognostic information that should feed into individualized treatment-intensity decisions [4,5]. In obstetric rheumatology, nationwide registry data continue to clarify how disease- and treatment-specific factors shape reproductive outcomes [6], while scoping reviews are progressively refining which targeted therapies are compatible with pregnancy and lactation [7]. Finally, artificial intelligence and automation are beginning to enter the personalization toolkit directly: a robotic ultrasound system paired with an AI scoring model has recently been shown to match expert rheumatologists in synovitis scoring according to EULAR-OMERACT criteria, foreshadowing a future in which imaging-based personalization tools of the kind discussed in this Special Issue could be further standardized and scaled [8]. It is against this rapidly evolving backdrop that we discuss the five contributions to this collection—not grouped in the order of their publication, but by the type of tool they employ to achieve personalization, moving from non-invasive imaging and clinimetric scores, through serological biomarkers, to invasive histopathological sampling.

Two contributions in this Special Issue show how non-invasive, standardized imaging and clinimetric assessments can support individualized patient stratification. Guedes et al. provide a comprehensive review of the ultrasonographic assessment of calcium pyrophosphate deposition disease (CPPD), tracing the evolution from early classification of deposition patterns to the OMERACT-standardized definitions that now underpin a reliable, reproducible diagnostic protocol. Their work underscores how ultrasonography—now incorporated into the 2023 ACR/EULAR classification criteria [9]—allows clinicians to characterize disease burden and joint involvement on a patient-by-patient basis, informing tailored diagnostic and therapeutic strategies.

De Angelis et al. take a complementary approach in SSc-associated interstitial lung disease (SSc-ILD), combining pulmonary function testing (DLco), patient-reported measures (modified Borg dyspnea scale, GERD-Q), and nailfold capillary density into a single diagnostic algorithm. By applying Bayes’ theorem through a Fagan nomogram, they translate four readily available, non-invasive measures into an individualized post-test probability of ILD—raising it from a pre-test value of 42% to a post-test value of 93% in their illustrative example. These findings resonate with the broader movement toward guideline-endorsed, non-invasive personalization tools, as reflected in the 2023 ACR/CHEST screening guideline for SSc-ILD [3], and with emerging AI-assisted ultrasound platforms that promise to further standardize operator-dependent imaging assessments of the kind used in CPPD [8].

A second group of contributions relies on laboratory and phenotypic data to anticipate disease trajectories and guide treatment choices. Benucci et al. investigated the factors associated with difficult-to-treat rheumatoid arthritis (D2T-RA) in a real-world cohort of 344 patients, identifying female sex, longer disease duration, higher rheumatoid factor and ACPA titers, elevated ESR, and glucocorticoid use as independent predictors of progression to a refractory phenotype. Notably, their data also point to a mechanistically grounded role for JAK inhibitors in achieving clinical stabilization in this hard-to-treat population. These results align with the 2025 EULAR RA management recommendations, which for the first time explicitly incorporate autoantibody status and acute-phase reactants as stratifying factors for early treatment escalation [1].

Giannakaki et al., in their narrative review on the management of rheumatic diseases in pregnancy, extend this same logic to a distinct clinical scenario. Across systemic lupus erythematosus, antiphospholipid syndrome, Sjögren’s syndrome, systemic sclerosis, and rheumatoid arthritis, they emphasize that predictive serological profiles and disease quiescence at conception are the strongest determinants of favorable maternal–fetal outcomes. Importantly, the authors also highlight persistent gaps in the field, including the underrepresentation of diverse populations in pregnancy studies and the absence of global pregnancy drug registries—gaps that recent nationwide registry data [6] and scoping reviews of targeted therapy safety [7] are only beginning to address, and that remain far from closed.

Not every tool for personalization is non-invasive, and not every study in this Special Issue reports an unambiguous benefit. Harada et al. examined the role of kidney biopsy in guiding treatment intensity in ANCA-associated vasculitis with renal involvement. Using propensity score overlap weighting in a cohort of 74 patients, they found that biopsy was associated with more intensive immunosuppressive therapy, yet—after adjustment—no statistically significant difference emerged in clinical outcomes such as end-stage kidney disease, death, or infectious complications within six months. This contribution is a valuable reminder that personalization is not unconditionally good: an invasive diagnostic tool may reshape therapeutic decisions without a demonstrable short-term benefit on hard outcomes, and the decision to biopsy must still be individualized against age, frailty, and the reversibility of renal lesions. This finding is consistent with ongoing efforts within the nephrology community to refine the prognostic yield of kidney biopsy—for instance through the 2024 KDIGO guideline and updated treatment standards, both of which aim to extract more individualized prognostic information from the same invasive procedure rather than simply expanding its indications [4,5].

Taken together, the five contributions collected here directly address several of the knowledge gaps identified in our original call: the need for standardized, reproducible imaging criteria (Guedes et al.); the integration of multiple non-invasive measures into a single diagnostic algorithm (De Angelis et al.); the individualization of biopsy-based decision-making (Harada et al.); the identification of biomarker-based predictors of refractory disease (Benucci et al.); and the synthesis of predictive risk factors in an understudied population of pregnant patients (Giannakaki et al.). At the same time, several gaps remain insufficiently addressed by the current literature, including by this Special Issue. First, external validation is lacking for most of the algorithms proposed here: De Angelis et al. explicitly note this limitation, and the CPPD ultrasound protocol summarized by Guedes et al. still shows only moderate inter-observer agreement at tendon and synovial sites. Second, the underrepresentation of diverse populations, already flagged by Giannakaki et al. in the obstetric setting, is a broader problem across rheumatology research: most of the predictive models discussed in this collection, and in the wider literature cited above, were derived from single-center, largely homogeneous cohorts [1,3,7]. Third, the cost-effectiveness of increasingly sophisticated personalized diagnostic pathways—whether a four-variable ILD algorithm or an AI-assisted ultrasound platform—remains essentially unstudied and will need to be addressed before such tools can be recommended for widespread adoption outside specialized centers [8].

Looking ahead, we highlight four priorities for future research in this field. First, prospective, multicenter validation of the diagnostic algorithms and risk scores proposed in this Special Issue and in the broader literature is needed before they can be adopted into routine guidelines, following the model already set by revised kidney risk scores in ANCA-associated vasculitis [4,5]. Second, the integration of artificial intelligence into imaging-based personalization—already demonstrated for RA synovitis scoring [8]—should be extended to other crystal arthropathies and connective tissue diseases, with attention to reproducibility across ultrasound machines and operators [10]. Third, larger and more inclusive pregnancy registries are needed to generate serological and clinical predictors that are valid across ethnic and socioeconomic groups, not only in the well-resourced settings where most current evidence originates [6,7]. Finally, the field would benefit from pragmatic, head-to-head trials comparing graduated, tool-based diagnostic strategies—starting non-invasive and escalating to invasive testing only when needed—against current standard practice, to establish whether the promise of personalization translates into better outcomes at a sustainable cost.

We are aware that the heterogeneity of the contributions collected here—spanning obstetric rheumatology, crystal arthropathy imaging, ANCA-associated vasculitis, systemic sclerosis, and rheumatoid arthritis—might at first appear to dilute the focus of this Special Issue. We would argue instead that this heterogeneity is itself a faithful reflection of the field: rheumatic diseases are inherently multisystemic, and their personalized management necessarily draws on an equally diverse toolkit of laboratory, imaging, clinimetric, and histopathological expertise. We hope readers will find this collection to present not a single, narrow theme, but a representative cross-section of how personalized medicine is being operationalized, tool by tool, across the breadth of contemporary rheumatology.

We warmly thank the authors for their valuable contributions and the reviewers for their rigorous and constructive evaluations, which substantially strengthened each of the papers in this collection. We look forward to the continued growth of personalized medicine research in rheumatic diseases.
